# Hepatic Encephalopathy in Children

**DOI:** 10.1007/s12098-023-04679-6

**Published:** 2023-06-13

**Authors:** Johanna Ascher Bartlett, Rohit Kohli

**Affiliations:** Department of Gastroenterology, Hepatology and Nutrition at Children’s Hospital Los Angeles, 4650 Sunset Blvd, MS 78, Los Angeles, CA 90027 USA

**Keywords:** Pediatrics, Cirrhosis, Liver failure, Encephalopathy

## Abstract

Hepatic encephalopathy, characterized by mental status changes and neuropsychiatric impairment, is associated with chronic liver disease as well as acute liver failure. In children, its clinical manifestations can be challenging to pinpoint. However, careful assessment for the development of hepatic encephalopathy is imperative when caring for these patients as progression of symptoms can indicate impending cerebral edema and systemic deterioration. Hepatic encephalopathy can present with hyperammonemia, but it is important to note that the degree of hyperammonemia is not indicative of severity of clinical manifestations. Newer forms of assessment are undergoing further research, and include imaging, EEG and neurobiomarkers. Mainstay of treatment currently includes management of underlying cause of liver disease, as well as reduction of hyperammonemia with either enteral medications such as lactulose and rifaximin, or even with extracorporeal liver support modalities.

## Introduction

Hepatic encephalopathy (HE) is defined as neuropsychiatric impairment and mental status changes that present in patients with liver disease [1, 2]. Patients with HE are at risk of developing cerebral edema, and its diagnosis and grading can be particularly challenging in pediatrics depending on a child’s age, developmental achievements, and easily confused symptoms. These patients require close monitoring while inpatient and may develop progressive or waxing and waning symptoms. Patients with acute liver failure or chronic liver disease may both be afflicted, though with slightly different presentations. Episodes of HE may be prompted by sepsis, upper gastrointestinal tract bleeding, dehydration, constipation, ileus, or small bowel obstruction, among other things [1, 3]. Degree of HE can be exacerbated by a variety of mechanisms including oxidative stress, hyperinflammatory states, altered cerebral blood flow and metabolic derangements. Best clinical care for these patients takes place in an experienced liver transplant center. This discussion will focus on the clinical presentation, grading and management of HE in children, as well as introduce areas of ongoing research.

## Pathogenesis of HE

The underlying mechanism of HE is not well-defined, but multiple theories have been proposed. One leading hypothesis surrounds gut-derived toxins and hyperammonemia, though other possibilities involve altered bile acids, cytokines, and chemokines [1, 4]. Ammonia is a byproduct of bacterial metabolism of both proteins and other nitrogenous compounds in the large intestine and is also generated during glutamine metabolism in small bowel enterocytes. A majority of ammonia is typically metabolized to an innocuous by-product (urea), during first-pass metabolism in the liver *via* the portal vein, which is then excreted [5]. As such, in the setting of liver disease which disrupts this natural blood flow, metabolism and excretion of ammonia is inhibited, leading to hyperammonemia and its increased passage across the blood-brain barrier [1]. In the brain, ammonia is postulated to cause cerebral edema by inducing swelling of type II astrocytes [1]. It is important to keep in mind that there is minimal, if any, clinical correlation between level of hyperammonemia and degree of HE. However, in the setting of extreme elevations, studies have shown increased risk of cerebral herniation when ammonia levels reach >200 μmol/L [4].

Additionally, accurate ammonia levels can be difficult to obtain. While arterial samples are the gold standard, these are not always feasible in the pediatric clinical setting. When collected as a venous sample, the level must be measured off of a specimen obtained from a free-flowing (without use of a tourniquet or clenching of the fist) blood draw, to minimize chances of a falsely elevated ammonia level due to excess release from skeletal muscle [1]. Finally, the specimen must be immediately placed on ice and transported to the lab to be processed within 20 min of collection. As such, there is high probability of invalid results which further highlights the caution clinicians should implement when assessing hyperammonemia to make clinical decisions.

## Clinical Considerations and Classification of HE

**Fig. 1:**
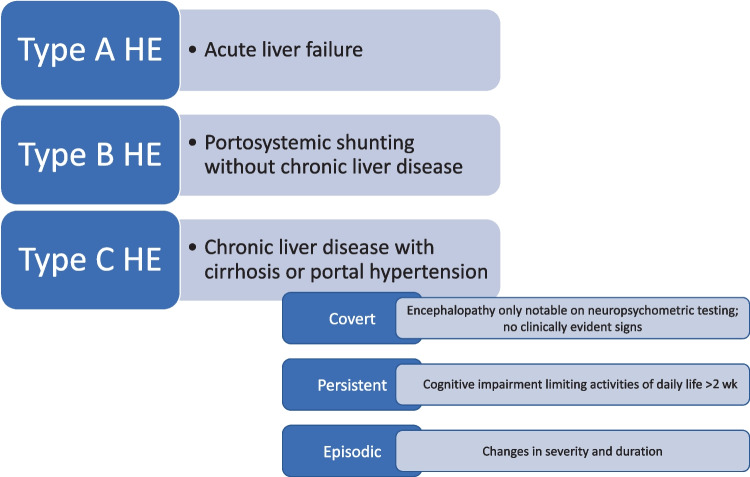
Classification of Hepatic Encephalopathy (HE) phenotypes.

HE associated with acute liver failure is known as Type A HE [1, 2]. Patients with HE due to portosystemic shunting but no underlying liver disease are categorized as Type B HE [1, 2]. Chronic liver disease with cirrhosis or portal hypertension, and HE, are known as Type C [1, 2]. Within Type C HE, there are several different forms: covert, persistent and episodic. Episodic encephalopathy changes in severity and duration; recurrent encephalopathy is defined as more than 2 episodes in one year; persistent encephalopathy is defined as cognitive impairment that limits activities of daily living for a period of more than 2 wk [1]. This classification system is summarized in Fig. 1.

Portosystemic shunts direct intestinal blood flow bypassing the liver, meaning neurotoxic intestinal metabolites that would have typically been metabolized by the liver are instead directed into the systemic circulation. These compounds can cross the blood-brain barrier, leading to clinical encephalopathy. In particular, patients who have undergone transjugular intrahepatic portosystemic shunt (TIPS) have been shown to develop HE at fairly high rates (~50%) [2]. However, TIPS is only used in the older adolescent population in the pediatric age group.

Patients with acute liver failure and encephalopathy are at higher risk of developing seizures and cerebral edema along with hyperammonemia than are patients with chronic liver disease. Of note, cerebral edema tends to be rarer in patients with chronic liver disease, possibly due to underlying cerebral atrophy [1]. Overall, patients with acute liver failure and HE have a higher risk of mortality and more often require emergent liver transplantation than do patients with chronic liver disease whose disease progression may be more subtle [4, 6]. For patients with acute on chronic liver disease, HE is often used as a diagnostic criterion for the acute episode of decompensation, and grade 3-4 HE may be associated with increased mortality in this population [7–9]. These patients, who present with sudden worsening of known underlying liver disease or cirrhosis as indicated by progression of ascites, gastrointestinal bleeding, cholangitis, infection or development of HE, often go on to develop multi organ system failure and progressive HE in the setting of acute on chronic liver failure [7, 8]. Children with acute on chronic liver failure have been known to experience increased mortality, but further research is needed to clarify the mechanisms and pathophysiology [7–9].

## Grading of Hepatic Encephalopathy

Standard grading of pediatric HE tends to rely on West Haven criteria **(**Table 1). The scoring systems have been established using adult data but are also adapted to the pediatric population. HE can be vague in children, with subtle clinical changes in mental status such as those listed in Table 2, still notable of progressive encephalopathic changes. Signs of pediatric HE may include irritability, fatigue, confusion, and can progress to coma (Table 2). As such, some of the signs of ongoing HE may initially be overlooked by the medical team as they can be attributed to expected child behavior in a hospital setting. In chronic liver disease, presentation may be more indolent as many patients have underlying cerebral atrophy.

**Table 1:** Grading of pediatric Hepatic Encephalopathy using West Haven Criteria

Grade of Encephalopathy	Neurological presentation (younger children, <4 y)	Neurological presentation (older children, >4 y)
Stage 1	Increased fussiness, sleep disturbance, easily distracted	Sleep disturbance, mood changes, mental fogginess
Stage 2	Increased fussiness, sleep disturbance, easily distracted	Progressive fatigue
Stage 3	Somnolence, agitation	Increased fatigue, confusion, speech abnormalities, hyperreflexia and increased tone
Stage 4 (coma)	Overt coma, decerebrate or decorticate posturing	Overt coma

**Table 2:**
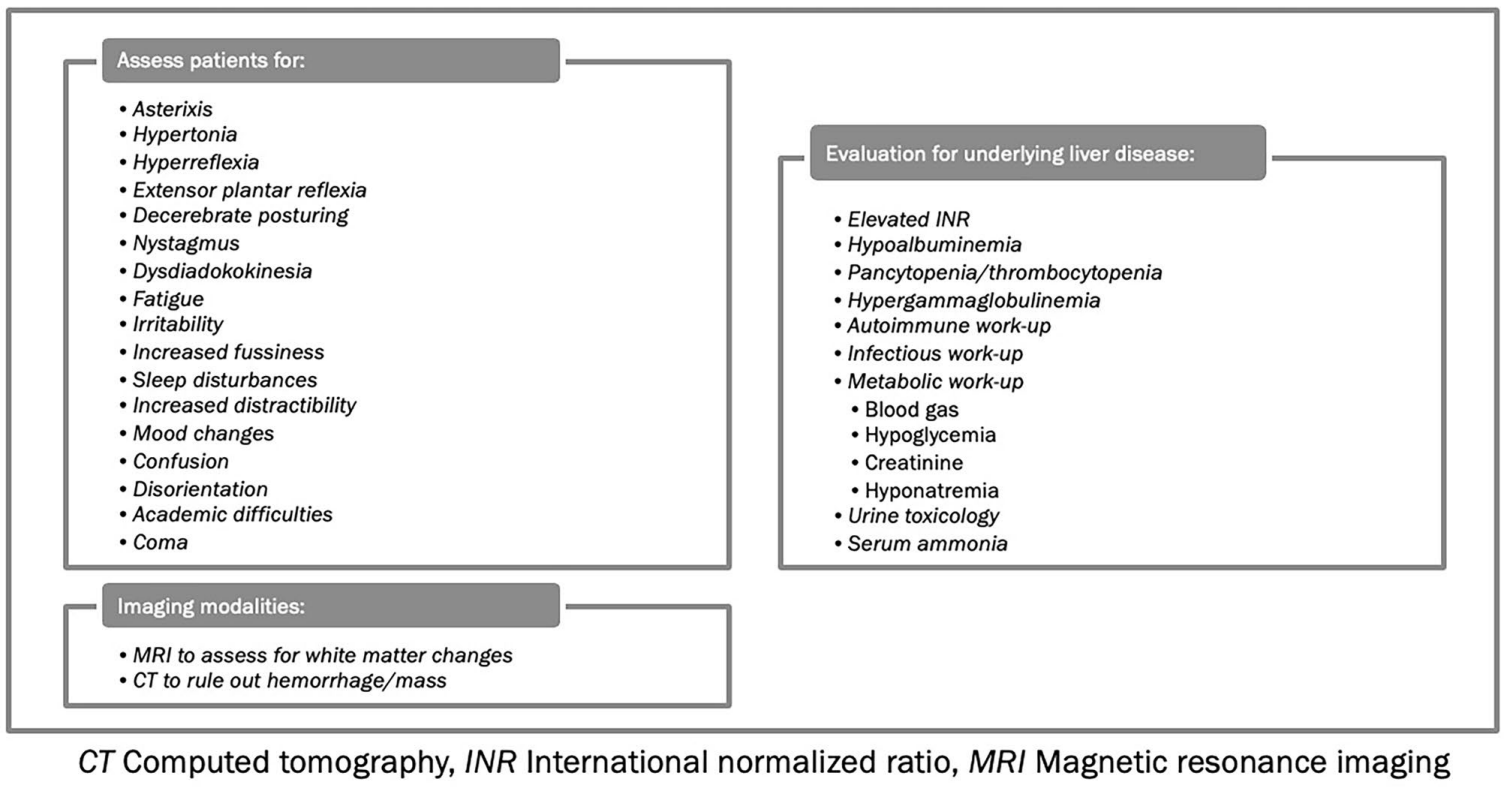
Assessment of pediatric Hepatic Encephalopathy

## Minimal Hepatic Encephalopathy

Minimal encephalopathy may also be known as grade 1 as scored with the West Haven Criteria. This degree of encephalopathy is known as covert encephalopathy, and presents with subtle mental status changes such as sleep disturbance, mood changes, anxiety, fatigue [3]. Yet when these patients are subjected to psychometric testing, their performance is abnormal [1]. These patients may even have normal mental status, and the only evidence of impairment is performance on psychometric testing [10]. This tends to be more common in patients with cirrhosis rather than other forms of liver disease, characterizing up to 50% of patients with chronic liver disease [10, 11]. Typically, minimal encephalopathy seems to affect executive functions rather than speech and can therefore be difficult to assess. In pediatric patients, this may mean these patients have more academic difficulties, and poor school performance [3]. Furthermore, biomarkers do not correlate with psychometric testing results.

## Management of Hepatic Encephalopathy

When evaluating a patient for HE, it is important to exclude other conditions that can present in a similar fashion. These conditions include but are not limited to respiratory failure, electrolyte derangements, status epilepticus, renal failure, stroke, hypoglycemia, central nervous system infections, metabolic acidosis and drug intoxication. Patients may also require evaluation of underlying liver disease, and workup should include a comprehensive metabolic panel with liver enzymes, complete blood count to assess for pancytopenia and thrombocytopenia, and coagulation panel with specific notation of international normalized ratio (INR) as described in Table 2. Further workup for the underlying etiology of liver disease is also important, and patients should be evaluated with autoimmune, infectious, and metabolic testing. Urine toxicology screening is needed to determine whether medication or drug effect is contributing to symptom presentation, and finally, a serum ammonia level to determine presence of hyperammonemia. Again, it is important to note that hyperammonemia does not correlate with the degree of clinical HE. Furthermore, collection of serum samples for ammonia level is very sensitive to technical variations and affects the results. This diagnostic approach is summarized in Table 2.

Overall, HE is a clinical diagnosis, and it can be difficult to clarify with concomitant concern for intensive care unit delirium. Imaging may be considered to exclude a cerebral mass or hemorrhage as confounding variables, and to evaluate for evidence of increased intracranial pressure (ICP) or cerebral herniation. Both magnetic resonance imaging (MRI) brain and computed tomography (CT) brain scans can be considered depending on the clinical context [1]. Furthermore, given the subtle but potentially progressive presentation of HE, close monitoring of the patient by bedside nursing, vigilant family/caregivers, and subsequent early intervention of the provider team if needed, is imperative. As such, these patients are best cared for in experienced liver transplant centers that are familiar with the signs and symptoms of HE, and understand this phase of disease can fluctuate, and have nuanced presentation as indicated in Table 1.

Treatment of HE largely depends on the underlying cause of liver disease. Mainstay of therapy tends to be supportive care, but there are medications to mitigate hyperammonemia as well (Table 3). Lactulose, a disaccharide which acidifies the intestinal lumen, is the most commonly used medication, and limits generation and absorption of gut ammonia. Lactulose stimulates passage of stool to eliminate ammonium converted by gut bacteria from ammonia [2, 10]. One study showed improvement in psychometric testing results in patients with minimal hepatic encephalopathy after treatment with lactulose [12]. Rifaximin may also be used, as this is a minimally absorbed broad-spectrum antibiotic that is solubilized by bile salts present in the small bowel to decrease ammonia production. Typically, lactulose is considered first-line therapy given its shorter time to onset than rifaximin [2, 4]. Other options for short-term treatment include empiric antibiotic therapy such as neomycin, metronidazole or oral vancomycin which all aim to alter the gut microbiome to reduce ammonia levels [2, 3, 10].

**Table 3:** Medical prevention of Hepatic Encephalopathy

Intervention	Dose	Side effects
Lactulose	0.5 mL/kg per dose • max 30 mL per dose for children and adolescents, up to TID for a total of 90 mL/d • 10 mL/d max. for infants • Titrate to achieve 2-3 stools per day with stool pH <6	Abdominal cramping, diarrhea, gassiness, abdominal distension, nausea, vomiting
Rifaximin	550 mg BID or 400 mg TID for patients >50 kg	Nausea, dizziness, fatigue, peripheral edema, pruritis, rash, diarrhea, joint pain

In the event of acute-onset progressive encephalopathy, patients should be closely monitored given the risk of increased ICP and cerebral edema. Management includes minimizing stimulation, elevating the head of the bed to 30 degrees, and prevention of further injury with the consideration of padded bed rails or a patient sitter. If the HE leads to cerebral edema, management of the associated increased ICP is indicated, and may include hyperventilation and hyperosmolar therapy with mannitol or hypertonic saline [4].

Dietary protein restriction is highly controversial in children of growing age, though intake of 1-2 g/kg/d has been suggested for patients with HE associated with acute liver failure and hyperammonemia, urea cycle defects, or chronic liver disease and acute onset HE in an effort to mitigate further ammonia production as a component of nitrogenous waste during protein catabolism [4, 10, 13, 14]. All sedative medications should be avoided, unless necessary for airway protective measures, especially benzodiazepines and opiate medications, which may alter the neurological exam further [3, 4]. Finally, extracorporeal liver support modalities may be required. Continuous renal replacement therapy (CRRT) treats HE by reducing hyperammonemia without inducing rapid fluid shifts, and removes water-soluble toxins [15]. Initiation of CRRT at Children’s Hospital Los Angeles (CHLA) is reserved for patients in liver failure with progressive encephalopathy grade 2 or higher, concerns for pulmonary edema, and hepatorenal syndrome that is unresponsive to medical management (Table 4). CRRT may also be considered for patients with hyperammonemia >100 μmol/L, or patients with renal failure and oliguria. It is important to note that recurrent HE in the setting of liver failure is an indication for liver transplantation.

**Table 4:** Indications for initiation of CRRT

Indications for CRRT
Evolving hepatic encephalopathy (grade 2 or higher)
Hepatic encephalopathy + clinical instability or metabolic derangements
Pulmonary edema due to large volume fresh frozen plasma (FFP) infusion
Hepatorenal syndrome unresponsive to diuretics
Blood ammonia level ≥100 *µ*mol/L
Na ≤130 mEq/L which is refractory to medical management

Adapted from the CHLA Liver Failure Protocol

*CRRT *Continuous renal replacement therapy

Prevention of HE in patients with chronic liver disease relies on limiting underlying disease progression. Further attempts may include avoiding hepatotoxic medications and reducing gastrointestinal bleeding risks in patients with known esophageal varices through endoscopic approach when possible [5].

## Newer Modalities of Investigation

Psychometric testing is limited in pediatrics and has mostly been validated in adult studies. Adapting adult assessments to children is difficult due to the variety of developmental achievements of children depending on their ages [3]. However, when appropriate, neuropsychiatric testing may be used to objectively measure severity of HE, especially in patients with chronic liver disease who are monitored in an outpatient setting [10].

In children with acute liver failure, EEG has been considered as a metric for monitoring degree of encephalopathy [16]. Patients who demonstrated moderate EEG changes (slowing and epileptiform discharges) were found to be more likely to require liver transplantation or die. In the same study, patients with low grade encephalopathy, <2, with normal or mild changes on EEG were more likely to recover without requirement of liver transplant [16].

New studies have attempted to identify neurobiomarkers that correlate more accurately with progression of encephalopathy than ammonia. One group which focused on patients with acute liver failure, noted that SB100β (an astrocyte marker) and IL-6, are present in higher amounts in patients with hepatic encephalopathy, which may highlight an inflammatory and immunologic component to hepatic encephalopathy [17]. Similarly, in patients with minimal HE and chronic liver disease, levels of IL-6 and TNF-α have been showed to be elevated [11].

## Conclusions

In summary, HE, which is a clinically-diagnosed phenomena associated with worsening liver failure (acute, chronic, or acute-on-chronic), can be an indicator of poor prognosis. HE is often initially managed conservatively with supportive care. When it progresses, a variety of interventions may be useful and range from medication to dietary changes to extracorporeal liver support systems. Yet HE may also present with subtle mental status changes and is often initially missed in the pediatric population. In some cases, patients with HE in the setting of chronic liver disease may only be identified with psychometric testing. Knowing this, it is imperative to manage children with liver failure in an experienced liver transplant center equipped with the appropriate treatment modalities to intervene when necessary, given the unpredictable and potentially progressive nature of HE.
